# Accuracy of probabilistic and deterministic record linkage: the case of tuberculosis

**DOI:** 10.1590/S1518-8787.2016050006327

**Published:** 2016-08-16

**Authors:** Gisele Pinto de Oliveira, Ana Luiza de Souza Bierrenbach, Kenneth Rochel de Camargo, Cláudia Medina Coeli, Rejane Sobrino Pinheiro

**Affiliations:** I Programa de Pós-Graduação em Saúde Coletiva. Instituto de Estudos em Saúde Coletiva. Universidade Federal do Rio de Janeiro. Rio de Janeiro, RJ, Brasil; II Instituto de Ensino e Pesquisa. Hospital Sírio-Libanês. São Paulo, SP, Brasil; III Instituto de Medicina Social. Universidade do Estado do Rio de Janeiro. Rio de Janeiro, RJ, Brasil; IV Instituto de Estudos em Saúde Coletiva. Universidade Federal do Rio de Janeiro. Rio de Janeiro, RJ, Brasil

**Keywords:** Tuberculosis, epidemiology, Data Accuracy, Sensitivity and Specificity, Epidemiological Surveillance, statistics & numerical data

## Abstract

**OBJECTIVE:**

To analyze the accuracy of deterministic and probabilistic record linkage to identify TB duplicate records, as well as the characteristics of discordant pairs.

**METHODS:**

The study analyzed all TB records from 2009 to 2011 in the state of Rio de Janeiro. A deterministic record linkage algorithm was developed using a set of 70 rules, based on the combination of fragments of the key variables with or without modification (Soundex or substring). Each rule was formed by three or more fragments. The probabilistic approach required a cutoff point for the score, above which the links would be automatically classified as belonging to the same individual. The cutoff point was obtained by linkage of the Notifiable Diseases Information System – Tuberculosis database with itself, subsequent manual review and ROC curves and precision-recall. Sensitivity and specificity for accurate analysis were calculated.

**RESULTS:**

Accuracy ranged from 87.2% to 95.2% for sensitivity and 99.8% to 99.9% for specificity for probabilistic and deterministic record linkage, respectively. The occurrence of missing values for the key variables and the low percentage of similarity measure for name and date of birth were mainly responsible for the failure to identify records of the same individual with the techniques used.

**CONCLUSIONS:**

The two techniques showed a high level of correlation for pair classification. Although deterministic linkage identified more duplicate records than probabilistic linkage, the latter retrieved records not identified by the former. User need and experience should be considered when choosing the best technique to be used.

## RESUMO

**OBJETIVO:**

Analisar a acurácia das técnicas determinística e probabilística para identificação de registros duplicados de tuberculose, assim como as características dos pares discordantes.

**MÉTODOS:**

Foram analisados todos os registros de tuberculose no período de 2009 a 2011 do estado do Rio de Janeiro. Foi desenvolvido algoritmo para relacionamento determinístico, usando conjunto de 70 regras, a partir da combinação de fragmentos das variáveis-chave com ou sem modificações (*Soundex* ou *substring*). Cada regra era formada por três ou mais fragmentos. Para a abordagem probabilística, foi necessário estabelecer ponto de corte para o escore, acima do qual os *links* seriam classificados automaticamente como pertencentes ao mesmo indivíduo. O ponto de corte foi obtido por meio do relacionamento da base de dados Sistema de Informação de Agravos de Notificação – Tuberculose com ela mesma, posterior revisão manual e curvas ROC e *precision-recall*. Foram calculadas a sensibilidade e especificidade para análise de acurácia.

**RESULTADOS:**

A acurácia variou de 87,2% a 95,2% para sensibilidade e 99,8% a 99,9% para especificidade para as técnicas probabilística e determinística, respectivamente. A presença de valores faltantes para as variáveis-chave e o baixo percentual da medida de similaridade para o nome e data de nascimento foram os principais responsáveis pela não identificação dos registros do mesmo indivíduo pelas técnicas utilizadas.

**CONCLUSÕES:**

As duas técnicas apresentam alta concordância para a classificação como par. Apesar de a técnica determinística ter identificado mais registros duplicados que a probabilística, a segunda recuperou registros não identificados pela primeira. A necessidade e a experiência do usuário devem ser consideradas para a escolha da técnica a ser utilizada.

## INTRODUCTION

Like other countries, Brazil has a large volume of health data collected via national information systems and available in different databases. Linkage between databases aims to identify if two or more records relate to the same entity, generally the same individual. This technique is used to identify duplications in a same file or between two or more files whose information must be gathered in a single database^12^. The absence of an univocal identifier field in databases prevents direct identification of records of a same individual in different databases, requiring the application of more sophisticated chaining algorithms based on the combination of identification variables. Probabilistic and deterministic record linkage are techniques used to this end^6^ ^,^ ^10^ ^,^ ^18^ ^,^ ^19^.

Probabilistic record linkage uses approximate comparison functions. Different weights are assigned to each field based on their discrimination power and vulnerability to error. Deterministic record linkage uses exact comparison functions and classification based on rules developed from the knowledge of specialists^6^. Specific computational routines must be developed for each problem. Low data quality, such the occurrence of missing data and typos, can contribute to the mismatch of variables, hence the importance of evaluating the accuracy of database linkage techniques.

The Ministry of Health is responsible for developing, managing and storing data from national health information systems. Although the systems are not automatically interconnected, it is possible to link these databases thanks to the existence of nominal identification variables with high discrimination power, especially when used in combination, which are standardized in the systems. Tuberculosis (TB) cases are compulsorily recorded in a national information system for disease notification (Notifiable Diseases Information System – Sinan-TB)^a^. The TB surveillance system provides different entries for each individual, since current regulation requires the notification of cases of recurrence and return following abandonment. However, there are incorrect duplicate records related to the same case, which must be identified and properly eliminated^1^ ^-^ ^3^ ^,^ ^b^.

Few studies compare the accuracy of linkage processes between secondary databases^15^. Moreover, the scientific literature seems to lack guidance on how to choose between existing techniques and comparison of their results.

The aim of this study was to analyze the accuracy of deterministic and probabilistic record linkage to identify TB duplicate records, as well as the characteristics of discordant pairs.

## METHODS

The study used information from the Sinan-TB database from 2009 to 2011 related to the state of Rio de Janeiro, available from the State Health Department. TB notifications terminated due to diagnosis change were excluded.

A data preprocessing phase was carried out to correct errors and standardize the content of the key variables (name; mother’s name; date of birth; address; and district) used in each technique. Data transformations included: removal of punctuation marks, accents, repeated blanks and prepositions; conversion of letters to uppercase; removal of numbers from variables intended to be exclusively composed of letters and vice versa; removal of terms indicating lack of information (doesn’t know, unknown, among others); replacement of double letters by a single one; standardization of date formats; standardization of address terms (“S.” was replaced by “Street,” “Av.” by “Avenue,” etc.).

Subsequently, new variables were generated from standardized mother’s name and address formats, with the performance of the following processes: 1) Parsing (separation of fragments into first name, second name, and so forth) and 2) Substringing (fragment parts such as “Maria” → “Mari;” “Oliveira” → “Veira”). Each new variable was also transformed into another containing its Soundex code^8^. This feature aims to transform the text into a phonetic code to homogenize small spelling differences (resulting from errors of pronunciation or understanding by the recorder), use or not of double consonants, among others.

The probabilistic approach involves standardizing, blocking and forming links (record pairs to be compared), applying comparison algorithms and generating similarity scores, setting thresholds to classify links into true pairs, non-pairs and doubtful pairs, manually reviewing doubtful pairs and removing duplications^7^ ^,^ ^c^.

The cutoff score, above which the links were classified as belonging to the same individual^5^ ^,^ ^c^, was obtained by linkage of the Sinan-TB database with itself, for a shorter period: 2010-2011. A single, more sensitive blocking strategy was used, with the Soundex of the first name and sex, to allow the largest possible number of record combinations. The links file was manually reviewed to classify them as pairs or non-pairs, i.e., as belonging or not to the same individual, respectively. The distribution of links classified as non-pairs ranged from -7.5 to 19.7, while that of pairs ranged from 6.9 to 34.9. The cutoff point was set at 18.3 based on the exploratory analysis of score distribution and the inspection of the ROC curves and precision-recall. The study used the ROCR library of the R statistical package version 2.15.3^17^.

To identify duplicate records, the same blocking strategy was used. The linkage variables were name, mother’s name and date of birth. The first two were compared using Levenshtein distance, and the third by using an exact algorithm^6^. For each link, a score given by the compound weight was calculated by the sum of the agreement or disagreement for each variable to be compared^9^ ^,^ ^c^. The score of 18.3 was applied for automatic classification and to form groups of records which were likely to belong to the same individual. The software used was OpenReclink, a widely employed program in health care with a specific routine to identify duplications^c^. The result of this routine is an input file plus a score and the group identification code. Therefore, it is no known which links were formed. For example, for a group composed of records A, B, C and D, it is not known if all records were linked with each other in the linkage process or if A linked with B and C, and B linked with D. A strategy was devised to rebuild those links: when there was no score equality of all records of the same group, the choice was to consider the record in the group with the highest score to form the link with the different score record.

A deterministic record linkage algorithm was developed in Stata 12.0, based on 70 rules, to identify the record groups of a same individual and classify them as a pair or non-pair. These rules were based on the key variables, as well as those created in the preprocessing phase. Some rules also used Soundex codes in a concatenated format (e.g., “Maria Antonia Santos” was represented by M600A535S532). In each rule, the content of three or more variables was compared exactly, regardless of the presence of data constraints (Table 1).

**Table 1:** Examples of rules used in the sequential algorithm.

Rule	Patient’s name	Mother’s name	Date of birth	Address	Constraints
1	Exact	Exact	Exact	-	No missing values Patient’s full name with at least 15 characters in length
2	Same Soundex (of full name)	Exact	Exact	-	No missing values Soundex composed of 3 or + parts No newborns or twins
3	Same Soundex (of full name)	Same Soundex (of full name)	Same day and year	-	No missing values Considering only uncommon names
4	Same four initial characters for first and middle names + exact Soundex for surname	Same Soundex (first name and surname)	Exact	Exact	No missing values No newborns or twins

The variable name was used in almost all rules, given its high discrimination power. Date of birth was used in some rules with information on day, month and year, while in others one or more of its components were used in combination. The variables city of residence or of notification, sex, date of notification, date of conclusion, notification number, notification unit code, as well as others related to place of residence (district, phone number, zip code and house number) were used in the original form. District was the only one of these variables to undergo preprocessing.

The rules were laid out sequentially, from those using fewer variables, with greater discrimination power, to those using several variables in combination, but which individually had less discrimination power. Discrimination power is understood as a variable’s capacity to independently discriminate an individual.

The inclusion of rules aimed to increase the algorithm’s sensitivity without losing specificity. With each new rule, new groups were found or new records added to existing groups, even though they did not pair with all the records already identified in the group. Rules that generated incorrect groups were modified or ultimately discarded following an extensive manual review.

Several data constraints were imposed in many rules. The universal constraint for all rules was that the groups could only be formed with records whose variables had no missing values. In addition, for certain rules which used a combination of variables with lower discrimination power, the content of some of those variables needed to have a minimum length of characters. Similarly, in some rules, the Soundex code had to have a minimum number of blocks (Maria Antonia Santos → M600-A535-S532 → 3 blocks).

Name infrequency was also considered in some rules. To this end, three separate databases were created indicating the frequency of the first, middle and last name in the Brazilian Mortality Information System (SIM) database for 2008-2010. Names appearing in the TB database but absent in SIM were considered rare. Records containing the same rare first, middle or last names were combined into groups, even when some of the other variables used in the rule had incomplete information. However, records with common rare names but with birth dates more than 20 years apart were not considered as belonging to the same group, since they could refer to father and son, for example. This possibility had to be considered, especially in the case of an infectious disease whose main transmission occurs within the household. The definition of rarity ranged from 500 to 1.000 for name frequency, according to the discrimination power of the other variables of the rule.

A manual review was carried out of the links classified as pairs by both techniques and of those with discordant classification. The classification of the manual review was considered the gold standard for calculations of sensitivity and specificity.

The following indicators were analyzed to profile records with discordant classification between the techniques:

1. Percentage of missing values for the variables name, mother’s name, sex, date of birth and address;
2. Score median of duplicate records identified by probabilistic linkage and not identified by deterministic linkage;
3. Percentage of same group records identified by probabilistic linkage with differences in the variable sex;
4. Similarity measure median for the variables name, mother’s name and date of birth of discordant records (calculated only for records with no missing values).

Levenshtein distance (LD) should be analyzed considering the size of the character string, as long strings are more likely to generate higher values^19^. A similarity measure (SM) was obtained by subtracting LD from the length of the longest full string and also dividing by this value. The result was multiplied by 100 to obtain the percentage and facilitate the analysis of results.

For groups with three or more records, the record link used to calculate SM was formed by the record presenting discordant classification between both techniques and the record which, in probabilistic linkage, had the highest score in the group. This alternative was adopted assuming that the record associated with the highest score would tend to be spelled more correctly. Records presenting difference in sex were excluded from the SM analysis, since they were not submitted to probabilistic linkage because they belonged to different logical blocks. Stata 12.0 software was used to analyze the records.

The project was approved by the Research Ethics Committee of the Institute of Studies in Collective Health of the Federal University of Rio de Janeiro (Process 114,604 of October 3, 2012).

## RESULTS

The Sinan-TB database contained 43,825 records. Table 2 shows the percentage of single records or of those forming groups of two to 10 records identified by deterministic and probabilistic linkage. In deterministic linkage, 78.7% were single records, and in probabilistic linkage the figure was 80.5%.

**Table 2:** Number and percentage of single records or by groups by deterministic and probabilistic record linkage. Sinan-TB, 2009-2011.

Records by group (n)	Deterministic record linkage		Probabilistic record linkage	
	
	Cases (n)	%	Cases (n)	%
1	34,506	78.7	35,266	80.5
2	6,944	15.8	6,546	14.9
3	1,502	3.4	1,280	2.9
4	548	1.3	468	1.1
5	215	0.5	160	0.4
6	66	0.2	60	0.1
7	35	0.1	35	0.1
9	9	0	-	-
10	-	-	10	0

Total	43,825	100	43,825	100

In deterministic linkage, 21.3% of the records were classified as pairs, and in probabilistic linkage, 19.5%. Discordant classification appeared in 1,812 records. In total, 527 records were classified as pairs by probabilistic linkage and not by deterministic linkage, and 1,285 records formed pairs by deterministic linkage and not by probabilistic linkage. The subsequent manual review, performed to determine the gold standard, showed no classification change for the group of records classified as pairs by both techniques (Table 3).

**Table 3:** Correlation analysis of record linkage techniques.

Probabilistic record linkage	Deterministic record linkage				

	Non-pair		Pair		Total
	
	n	%	n	%	
Non-pair	33,980	96.4	1,285	3.6	35,265
Pair	527	6.2	8,033	93.8	8,560

Total	34,507	78.7	9,318	21.3	43,825

In the accuracy analysis, sensitivity and specificity values for deterministic linkage were 95.3% and 99.9%, respectively. For probabilistic linkage the figures were 87.2% and 99.8%, respectively (Table 4).

**Table 4:** Sensitivity and specificity analysis of record linkage techniques.

Standard	Total	Deterministic record linkage		Probabilistic record linkage	
	
		Pair	Non-pair	Pair	Non-pair
Pair	9,741	9,283	458	8,491	1,250
Non-pair	34,084	35	34,049	69	34,015
Total	43,825	9,318	34,507	8,560	35,265
Sensitivity (95%CI)		95.3	(94.8–95.7)	87.2	(86.5–87.8)
Specificity (95%CI)		99.9	(99.8–99.9)	99.8	(99.7–99.8)

Of the records classified as pairs by deterministic linkage and non-pairs by probabilistic linkage, none had missing values for the variables name and sex, 5.3% had missing values for mother’s name, 6.3% for date of birth and 0.5% for address. Ten percent of the records presented missing values for at least one of these variables. The score median of records classified as pairs by probabilistic linkage and as non-pairs by deterministic linkage was 24.2, ranging from 20.3 to 32.3. No records were found with missing information for the variables name and sex. For mother’s name, 14.4% of records had missing values; for date of birth, 11.0%; and for address, 3.8%. More than a quarter of the records had missing values for at least one of the analyzed variables.

Of the 733 links classified as pairs by deterministic linkage and non-pairs by probabilistic linkage, 15.7% showed differences for the variable sex. Approximately 26.0% had SM lower than 70.0% for the variable name, 26.6% for mother’s name, and 8.3% for date of birth. For links classified as pairs only by probabilistic linkage, 16.6% had SM lower than 70.0% for the variable mother’s name and 10.6% for date of birth. As to the SM median, the biggest difference between the groups was for the variable name (81.6 for pair links by deterministic linkage and 100 for pair links by probabilistic linkage) (Table 5).

**Table 5:** Characteristic of records with discordant classification by deterministic and probabilistic record linkage.

Record characteristics	Pair by deterministic and non-pair by probabilistic linkage		Pair by probabilistic and non-pair by deterministic linkage	
	
	(N = 1,285)		(N = 527)	
			Median	CI95%
Score	-	-	24.2	20.3–32.3

	n	%	n	%
Missing sex value	0	0	0	0
Missing name value	0	0	0	0
Missing mother’s name value	68	5.3	76	14.4
Missing date of birth value	81	6.3	58	11.0
Missing address value	7	0.5	20	3.8
Combined: unknown mother’s name; or unknown date of birth; or unknown address	129	10.0	141	26.7

Link characteristics	**Pair by deterministic and non-pair by probabilistic linkage^a,b^**		**Pair by probabilistic and non-pair by deterministic linkage^a,b^**	
		
	(N = 733)		(N = 293)	

	n	%	n	%
Difference in sex	115	15.7	0	0
Similarity measure for name lower than 70,0%	160	25.9	0	0
Similarity measure for mother’s name lower than 70,0%	147	26.6	36	16.6
Similarity measure for date of birth lower than 70,0%	45	8.3	25	10.6

	Median	IC95%	Median	IC95%

Similarity measure for name x 100	81.6	69.4–94.4	100	95.0–100
Similarity measure for mother’s name x 100	91.3	68.2–100	94.4	85.7–100
Similarity measure for date of birth	100	10.0–100	87.5	75.0–100

^a^ Calculating Levenshtein distance and assessing the difference in sex between records required comparing the records of the record group of the same patient. Comparing discordant records: (i) when the two discordant records were identified by only one of the techniques, the calculation was done between them; (ii) when they were records identified by both techniques and only one of them was not identified by one of the techniques, the calculation was done by comparing the unidentified record with the record of highest score in the group.

^b^ For this calculation, the group of records that were blocked by sex or had missing information for one of the variables were excluded.

## DISCUSSION

The two techniques showed a high correlation in classifying records as pairs. However, deterministic linkage retrieved 8.8% more records than probabilistic linkage, despite the latter having identified records that were not identified by the former. Although the specificity of both techniques was similar, sensitivity was higher in deterministic linkage, corroborating the findings of other studies using the Sinan database^8^ ^,^ ^15^. The sensitivity and specificity values for probabilistic linkage in this study are consistent with those presented in other studies^9^ ^,^ ^12^ ^,^ ^13^ ^,^ ^16^.

The occurrence of variables with missing values and SM lower than or equal to 70.0% for key variables was the main reason for not identifying pairs in both techniques. Additionally, blocking by sex in probabilistic linkage was also responsible for the failure to form certain groups. Removing the variable sex from the initial blocking to set the cutoff point might minimize this problem, but greatly increase the volume of links to be manually reviewed, because the blocking key would become even more sensitive. On the other hand, the manual review would be performed only once and the established cutoff point would be applied to works with the same database. Although there are no missing values for the variable name, the lack of information for the variables mother’s name and date of birth in Sinan-TB is still significant^2^. Improving the quality of TB data is a constant challenge in Brasil^1^ ^,^ ^13^ ^,^ ^17^. This problem is reflected in the epidemiological and performance indicators, masking the real situation of TB in the country, which skews the analyses needed for decision-making and developing new control strategies^1^ ^,^ ^2^ ^,^ ^11^ ^,^ ^14^.

Variables related to address were used as auxiliary criteria in classifying records by deterministic linkage. Even though this variable should be used with caution, since patients might change their address between notifications, it assists in the decision. In probabilistic linkage between different databases, these variables can be used in the manual review to support classification. In this study, we chose not to perform the manual review after implementing the routine to identify duplications. In deterministic linkage, a different treatment was considered for common names in the algorithm. The influence of these strategies in retrieving pairs was not investigated, but they are believed to have influenced classification. The strategy of matching by frequency (information on name infrequency) could be incorporated into the probabilistic linkage software, minimizing the occurrence of false positives in high scores due to the presence of homonyms.

The high-score median for records classified as pairs by probabilistic linkage and non-pairs by deterministic linkage indicates that mutually similar key variables were not identified. This attests the need to improve the deterministic routine.

The deterministic algorithm used commercial statistical software whose interface is in English and requires prior knowledge of users of the programming language used by the software. In addition, the adaptation of the algorithm to databases other than Sinan-TB is conditional on knowledge of these databases and programming experience. Transcribing it using free software might facilitate its wider use. Probabilistic linkage used free national software which also requires prior knowledge. However, as the OpenReclink routine to identify duplications allows automatic classification of groups, it is easier to use^4^ ^,^ ^c^.

The study did not measure time and difficulties in using each technique. However, both the elaboration of the deterministic algorithm and the manual revision to set the cutoff point for probabilistic linkage demanded a considerable amount of time and experience from the researchers.

Sinan has specific intrinsic routines to identify and treat duplicate records^15^. However, its algorithm uses the variable name with no previous correction, which reduces its efficiency. Additional limitations include the large number of records, absence of feedback from national level to other levels, and the need for local computer networks with good quality and speed. Due to the high number of annual notifications, treatment of duplications at national level must be done “outside” Sinan, requiring the application of linkage techniques at each data transfer between computerized levels^a^.

The definition of which technique to use to identify duplicate records in Sinan-TB depends on the purpose and need of users. As the search for methodological perfection is a premise in research, deterministic linkage may be used initially to retrieve most of the links, followed by probabilistic linkage to retrieve the links that were not found by the former. In routine TB control programs at the three levels of government, removal of duplication is performed several times a year, which is extremely time-consuming for the professionals involved. The use of deterministic linkage is recommended provided the prerequisites for its use are met. Otherwise, probabilistic linkage is indicated, which, if necessary, can be rendered more sensitive with the performance of a manual review of the groups with scores below the set cutoff point, to retrieve new links previously unidentified. As for the construction of new deterministic routines and improvement of existing ones, probabilistic linkage, being generic, should be used as an initial strategy to accumulate knowledge to be incorporated in the development of deterministic linkage. Such accumulation of knowledge is important, since deterministic linkage is less specific and might generate far less accurate results than those obtained by probabilistic linkage. For Grannis et al., although the sensitivity of deterministic linkage may reach 100%, it can be considerably reduced for data with different characteristics of identification (ethnic names, for example), and therefore probabilistic linkage is considered to be more efficient^9^. Probabilistic linkage, on the other hand, can increase its accuracy even further by incorporating new variables or strategies to improve comparison and classification of links.

Sinan undergoes routine updates to meet the needs of users and of the national surveillance system^a^. This study contributes to the discussion and incorporation of new decision strategies for duplication removal. It also supports researchers and professionals of the Brazilian Unified Health System in choosing the best linkage technique to use. Given the significant contributions and good results generated by the use of database linkage in the health sector, it is important that further studies on record linkage accuracy be carried out in Brazil.

## Footnotes

a Ministério da Saúde, Secretaria de Vigilância em Saúde, Departamento de Vigilância Epidemiológica. Sistema de Informação de Agravos de Notificação (SINAN): normas e rotinas. 2.ed. Brasília (DF); 2007.

b Ministério da Saúde, Secretaria de Vigilância em Saúde. Manual de recomendações para o controle da tuberculose no Brasil. Brasília (DF); 2011. (Série A. Normas e Manuais Técnicos).

c Camargo Jr KR, Coeli CM. OpenReclink: guia do usuário [cited 2015 Jun 5]. Available from: http://reclink.sourceforge.net/
